# Impact of Coronavirus Disease 2019 on Sick Sinus Syndrome: Trends in Incidence and Pacemaker Implantation

**DOI:** 10.19102/icrm.2025.16102

**Published:** 2025-10-15

**Authors:** Dharmindra Dulal, Ahmed Maraey, Blair Grubb

**Affiliations:** 1 Department of Internal Medicine, Ohio State University Wexner Medical Center, Columbus, Toledo, OH, USA; 2 Division of Cardiovascular Medicine, University of Toledo Medical Center, Toledo, OH, USA

**Keywords:** COVID-19, incidence rate, pacemaker implantation, sick sinus syndrome

## Abstract

Sick sinus syndrome (SSS) is a cardiac conduction disorder that often necessitates pacemaker implantation, especially in older adults. Emerging evidence suggests a potential association between coronavirus disease 2019 (COVID-19) infection and SSS, but the impact on SSS trends and permanent pacemaker (PPM) implantation rates remains unclear. This study compares the pre- and post–COVID-19 trends in SSS incidence and PPM implantation rates. Using the TriNetX Research Network, we analyzed the monthly incidence rate (IR) of SSS and the rate of PPM implantation in the overall population from January 2018 to December 2023. Additionally, we conducted a subgroup analysis focusing on patients >50 years of age to examine trends in IR and PPM implantations during the same period. To evaluate changes before and after COVID-19, we used interrupted time series analysis, with March 1, 2020, as the cutoff. In the overall SSS population, the IR increased significantly post–COVID-19 (IR, 1.80 cases/100,000 person-years [PY] per month; *P* < .001), which was accompanied by a significant rise in PPM implantation rates (119.16 cases/100,000 PY per month; *P* < .001). Among patients <50 years of age, the IR increased post–COVID-19 (IR, 0.355 cases/100,000 PY per month; *P* < .001), but PPM implantation rates in this subgroup remained unchanged (*P* = .897). Our findings suggest an increase in SSS incidence across all age groups post–COVID-19. However, the lack of increased PPM implantation in younger patients may reflect either a more transient disease course or a higher threshold for device implantation in this age group. Further research is needed to determine the prognosis of SSS in the recent era.

## Introduction

Sick sinus syndrome (SSS) is a heart rhythm disorder resulting from the malfunction of the sinoatrial (SA) node.^1^ This condition leads to various arrhythmias, including sinus bradycardia, SA block, sinus arrest, and tachycardia–bradycardia syndrome, and is associated with symptoms such as dizziness, syncope, palpitations, and fatigue.^2^ The etiology of SSS is multifactorial, encompassing age-related degenerative fibrosis, infiltrative diseases, medications, metabolic disturbances, and autonomic dysfunction.^1,3^ The prevalence of SSS increases with age, affecting approximately 1 in 600 patients >65 years of age, and is the leading cause of permanent pacemaker (PPM) implantation in elderly populations.^4^

The emergence of the coronavirus disease 2019 (COVID-19) pandemic has introduced new challenges in understanding cardiovascular complications associated with COVID-19. Recent studies have reported instances of bradyarrhythmia, including SSS, in patients with COVID-19.^5–7^ The mechanisms by which COVID-19 may induce SSS are multifactorial, encompassing direct invasion of myocardial cells and the conduction system via angiotensin-converting enzyme 2 (ACE2) receptors.^8,9^ Additionally, the systemic inflammatory response and cytokine storm can exacerbate myocardial injury and disrupt normal cardiac conduction.^8–10^ Hypoxia resulting from respiratory complications may further impair SA node function and contribute to arrhythmias.^11^

Given these observations, it is crucial to investigate the impact of COVID-19 on the incidence of SSS and PPM implantation trends. This study analyzes and compares pre- and post–COVID-19 SSS incidence rates, then performs an assessment of trends in PPM implantation in this population. Additionally, a subgroup analysis of patients <50 years of age was conducted to determine whether SSS incidence increased similarly in younger individuals and whether this group experienced a corresponding rise in PPM implantation or exhibited distinct disease progression and management patterns.

## Methods

We conducted an observational study using the TriNetX Research Network, which provides access to electronic health records of over 115 million patients from >80 health care organizations, primarily in the United States. The dataset includes aggregate, de-identified information in compliance with the de-identification standard outlined in section §164.514(a) of the Health Insurance Portability and Accountability Act Privacy Rule. All data were collected using International Classification of Diseases, Tenth Revision, codes. As only de-identified data were used, institutional review board approval was not required.

We analyzed the monthly incidence rate (IR) of SSS in a cohort of 60,548,053 patients from January 1, 2018, to December 31, 2023. Additionally, we examined PPM implantation rates among SSS patients (n = 208,628) during the same period. To explore age-related differences, we conducted a subgroup analysis in patients <50 years of age drawn from a cohort of 31,647,682 individuals. Within this subgroup, we further examined PPM implantation trends in those diagnosed with SSS (n = 14,290). To evaluate the impact of COVID-19 on SSS and PPM implantation trends, we performed an interrupted time series (ITS) analysis using R version 4.4.1 (The R Project for Statistical Computing, Vienna, Austria), with March 1, 2020, as the cutoff to define pre- and post–COVID-19 periods. ITS allowed us to account for pre-existing trends and to assess changes in incidence and prevalence. In all analyses, IR is defined as cases per 100,000 person-years (PY), with all estimates reported as monthly rates.

**Figure 1:**
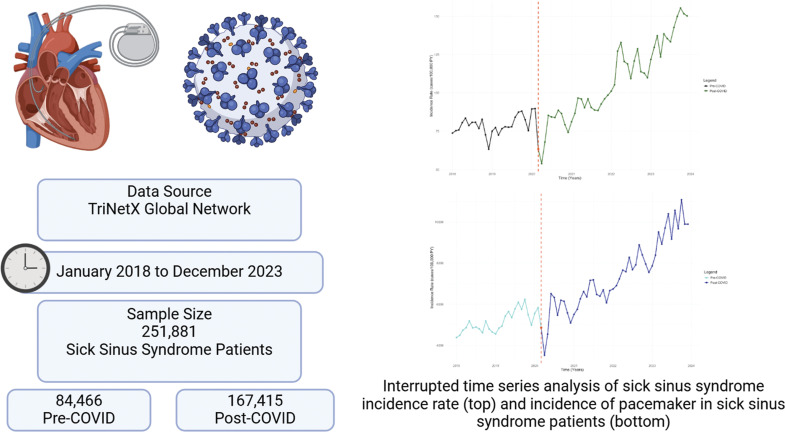
Key details of the study methods.

See **Figure 1** for key methods details.

## Results

### Baseline characteristics

A total of 84,466 patients with SSS were included in the pre–COVID-19 cohort and 167,415 were included in the post–COVID-19 cohort. The mean age was slightly higher post–COVID-19 (71.9 ± 12.4 vs. 70.5 ± 11.9 years; *P* < .0001), while racial and gender distributions were similar (*P* > .05). The post–COVID-19 cohort had higher prevalence rates of primary hypertension (68.77% vs. 64.28%), atrial fibrillation/flutter (49.77% vs. 48.10%), chronic ischemic heart disease (42.45% vs. 39.51%), type 2 diabetes mellitus (31.03% vs. 28.36%), and systolic heart failure (13.44% vs. 11.21%) (*P* < .0001 for all). Atrioventricular (AV) block was also more common post–COVID-19 (first degree, 9.44% vs. 7.38%; second degree, 5.65% vs. 4.96%; complete AV block, 9.24% vs. 8.79%; *P* < .001). Medications like β-blockers, anti-arrhythmics, and calcium channel blockers were prescribed more frequently in the post–COVID-19 cohort (*P* < .0001 for all) **(Table 1)**.

**Table 1:** Comparison of Baseline Characteristics of Patients with Sick Sinus Syndrome Before and After Coronavirus Disease 2019

Categories	Variables	Pre–COVID-19 (n = 84,466)	% Pre-COVID	Post–COVID-19 (n = 167,415)	% Post-COVID	*P* Value
Demographics	Age at index (years)	70.5 ± 11.9	100%	71.9 ± 12.4	100%	<.0001
	White	62,871	74.43%	124,861	74.58%	.4202
	Male	44,036	52.14%	87,644	52.35%	.3038
Diagnoses	Primary hypertension	54,298	64.28%	115,133	68.77%	<.0001
	Atrial fibrillation and flutter	40,630	48.10%	83,329	49.77%	<.0001
	Chronic ischemic heart disease	33,372	39.51%	71,066	42.45%	<.0001
	Type 1 diabetes mellitus	3202	3.79%	5372	3.21%	<.0001
	Type 2 diabetes mellitus	23,954	28.36%	51,945	31.03%	<.0001
	Chronic kidney disease	16,694	19.76%	40,734	24.33%	<.0001
	Cardiomyopathy	11,663	13.81%	24,584	14.68%	<.0001
	Diastolic heart failure	11,380	13.47%	28,181	16.83%	<.0001
	Systolic heart failure	9470	11.21%	22,493	13.44%	<.0001
	Atrioventricular block, first degree	6237	7.38%	15,807	9.44%	<.0001
	Atrioventricular block, second degree	4192	4.96%	9460	5.65%	<.0001
	Atrioventricular block, complete	7422	8.79%	15,476	9.24%	.0002
	Systemic connective tissue disorders	2448	2.90%	5803	3.47%	<.0001
Medications	β-Blockers	46,318	54.84%	101,139	60.41%	<.0001
	Anti-arrhythmics	42,282	50.06%	99,928	59.69%	<.0001
	Calcium channel blockers	30,643	36.28%	71,166	42.51%	<.0001
	Digitalis glycosides	6086	7.21%	11,526	6.89%	.0029
Lab data	Blood pressure, systolic	128 ± 20.4		129 ± 21.2		<.0001
	Blood pressure, diastolic	71.3 ± 12.2		71.3 ± 12.3		.749
	Sodium (moles/volume) in serum, plasma, or blood	139 ± 3.25		139 ± 3.32		.361
	Potassium (moles/volume) in serum, plasma, or blood	4.23 ± 0.478		4.22 ± 0.486		.225
	Hemoglobin (mass/volume) in blood	12.9 ± 2.06		12.8 ± 2.12		.193
	BMI (kg/m^2^)	29.6 ± 6.82		29.4 ± 6.82		.518
	Magnesium (mass/volume) in serum, plasma, or blood	2 ± 0.304		1.99 ± 0.295		.22
	Hemoglobin A1c/hemoglobin, total in blood	6.4 ± 1.59		6.31 ± 1.51		<.0001
	Corrected QT interval	450 ± 42.3		450 ± 43.1		.7687
	Left ventricular ejection fraction (%)	56.3 ± 13.2		56.4 ± 13.1		.7533

*Abbreviations:* BMI, body mass index; COVID-19, coronavirus disease 2019; SSS, sick sinus syndrome.

### Overall trends in sick sinus syndrome and permanent pacemaker implantation

**Figure 2:**
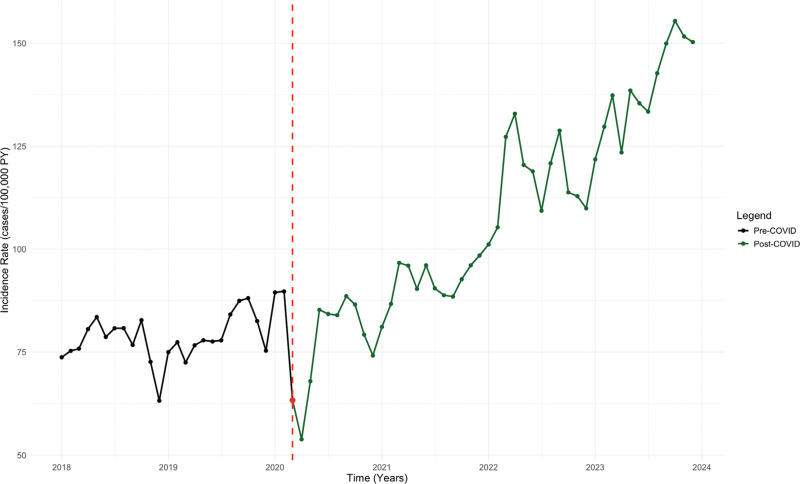
Interrupted time series analysis of the sick sinus syndrome incidence rate. *Abbreviations:* COVID, coronavirus disease; IR, incidence rate; ITS, interrupted time series; PY, person-years; SSS, sick sinus syndrome.

**Figure 3:**
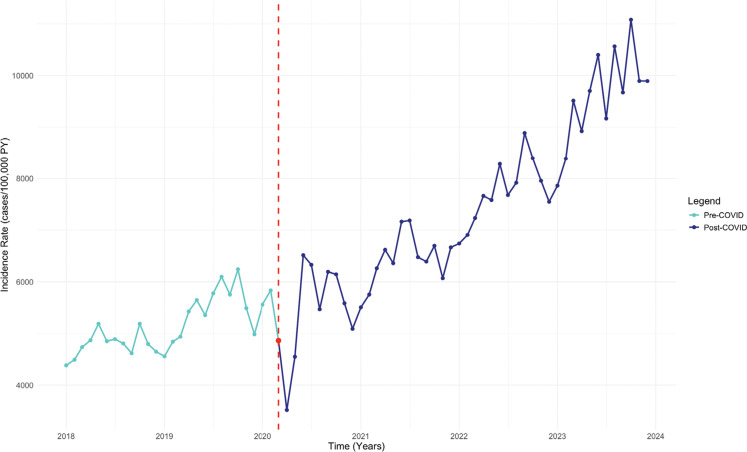
Interrupted time series analysis of the pacemaker placement incidence rate in patients with sick sinus syndrome. *Abbreviations:* COVID, coronavirus disease; IR, incidence rate; ITS, interrupted time series; PY, person-years; SSS, sick sinus syndrome.

The IR of SSS was stable during the pre–COVID-19 period (estimate, monthly increase of 0.34 cases/100,000 PY; 95% confidence interval [CI], −0.04 to 0.74; *P* = .091). Post–COVID-19, the IR increased significantly to 1.80 cases/100,000 PY per month (95% CI, 0.97–2.62; *P* < .001) **(Figure 2 and Table 2)**. The rate of PPM implantation in SSS patients had a significant upward trend during the pre–COVID-19 period, with an increase of 51.18 cases/100,000 PY per month (95% CI, 22.36–80.00; *P* = .001). This trend skyrocketed post–COVID-19, with a total increase of 119.16 cases/100,000 PY per month (95% CI, 59.04–179.30; *P* < .001) **(Figure 3)**.

**Table 2:** Interrupted Time Series Analysis of Sick Sinus Syndrome and Pacemaker Implantation Trends Before and After Coronavirus Disease 2019

Categories	Variables	Term	Estimate	Standard Error	*T* Value	CI	*P* Value
Overall SSS cases	IR	Pre-COVID	0.34	1.99E−01	1.72	−0.04 to 0.74	9.07E−02
		Post-COVID	1.80	2.16E−01	6.74	0.97 to 2.62	4.09E−09
	Pacemaker IR	Pre-COVID	51.18	14.44	3.54	22.36 to 80.0	7.00E−04
		Post-COVID	119.16	15.69	4.33	59.04 to 179.3	4.97E−05
SSS patients aged <50 years	IR	Pre-COVID	7.40E−03	7.08E−02	0.10	−0.13 to 0.15	9.17E−01
		Post-COVID	0.36	7.70E−02	4.61	−9.6E−05 to 0.00038	1.82E−05
	Pacemaker IR	Pre-COVID	12.03	18.44	0.65	−24.77 to 48.82	5.17E−01
		Post-COVID	14.64	20.04	0.13	−62.14 to 91.42	8.97E−01

*Abbreviations:* CI, confidence interval; COVID, coronavirus disease; IR, incidence rate; ITS, interrupted time series; SSS, sick sinus syndrome.

### Subgroup analysis

#### Sick sinus syndrome and permanent pacemaker implantation in patients <50 years of age

**Figure 4:**
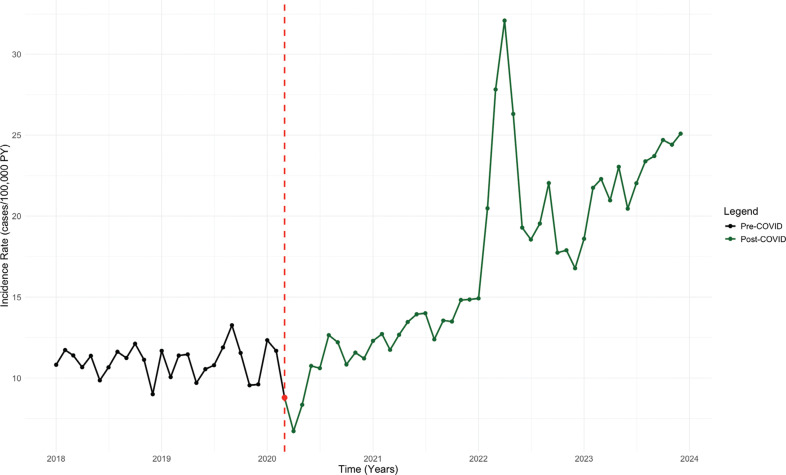
Interrupted time series analysis of sick sinus syndrome incidence rate in patients aged <50 years. *Abbreviations:* COVID, coronavirus disease; IR, incidence rate; ITS, interrupted time series; PY, person-years; SSS, sick sinus syndrome.

**Figure 5:**
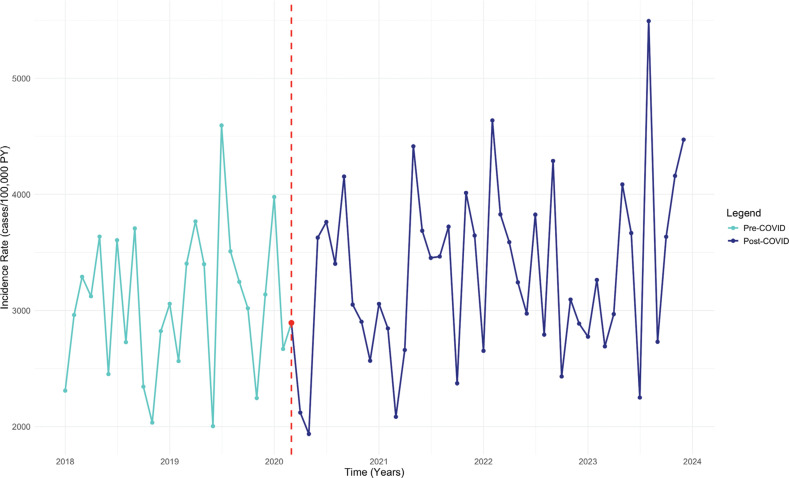
Interrupted time series analysis of pacemaker placement incidence rate in patients with sick sinus syndrome aged <50 years. *Abbreviations:* COVID, coronavirus disease; IR, incidence rate; ITS, interrupted time series; PY, person-years; SSS, sick sinus syndrome.

In patients <50 years of age, we found no significant increase in the IR of SSS pre–COVID-19 (estimate, 0.007 cases/100,000 PY per month; 95% CI, −0.13 to 0.15; *P* = .917). However, after the pandemic, a significant upward trend was noted in IR (0.362 cases/100,000 PY per month; 95% CI, 0.067–0.66; *P* < .001) **(Figure 4)**. There were no significant trends in PPM implantation rates in patients <50 years of age pre–COVID-19 (12.03 cases/100,000 PY per month; 95% CI, −24.77 to 48.82; *P* = .517) or post–COVID-19 (14.64 cases/100,000 PY per month; 95% CI, −62.14 to 91.42; *P* = .897) **(Figure 5)**.

## Discussion

In this extensive retrospective real-world study, we highlight key findings on the impact of COVID-19 on the incidence of SSS and PPM implantations. Our analysis reveals a significant post-pandemic increase in the incidence of SSS in the overall population, alongside a notable rise in PPM implantations among SSS patients. Similarly, in our subgroup analysis of patients <50 years of age, we observed a stable IR pre-pandemic that significantly increased post–COVID-19. However, this rise in SSS among younger patients (<50 years) was not accompanied by a corresponding increase in the number of PPM implantations.

Since the onset of the COVID-19 pandemic, multiple case reports and case series have documented new-onset SSS in patients infected with severe acute respiratory syndrome coronavirus 2 (SARS-CoV-2). Peigh et al. described two elderly patients who developed sinus bradycardia during acute infection, with bradyarrhythmias persisting for weeks post-infection.^12^ Cimino et al. reported a 34-year-old man with COVID-19 who experienced prolonged asystole due to sinus node dysfunction (SND),^6^ while another case involved a previously healthy 51-year-old patient who developed sinus arrest during COVID-19–related acute hypoxic respiratory failure.^7^ Similarly, Gatto et al. presented a case series of multiple patients with bradyarrhythmias, including SSS.^5^ These cases, along with our study’s findings, suggest that COVID-19 may serve as a trigger for SSS, leading to persistent or transient bradyarrhythmias in affected individuals.

The mechanisms underlying SARS-CoV-2–induced SSS are complex. Studies indicate that the virus’s strong affinity for ACE2 receptors, which are highly expressed in cardiac tissue, facilitates direct myocardial invasion.^8,9^ This invasion triggers myocardial inflammation and fibrosis, disrupting the electrical conduction system and contributing to arrhythmias. Additionally, the systemic inflammatory response and cytokine storm further exacerbate myocardial injury and impair SA node function.^8–10^ Furthermore, a hamster model study by Han et al. demonstrated that COVID-19 can directly infect SA node pacemaker cells, inducing ferroptosis and leading to SA node dysfunction.^13^

SSS is a leading cause of PPM implantation in the United States.^4,14^ Given the rise in SSS post–COVID-19, we investigated whether this trend corresponded to an increase in PPM placement. Our analysis revealed a significant post–COVID-19 increase in PPM implantation rates among SSS patients compared to the pre-pandemic period. These findings align with results of previous studies, such as that by Elices-Teja et al., who documented one of the earliest cases of COVID-19–induced SSS requiring PPM implantation in a 75-year-old woman.^15^ Similarly, Tovia-Brodie et al. conducted a multinational study showing that 18.7% of patients diagnosed with SSS after COVID-19 required PPM implantation.^16^

However, we found no significant increase in PPM implantation among younger patients (<50 years) with SSS post–COVID-19. This disparity may be due to the transient nature of SSS in younger individuals, who may have a higher likelihood of spontaneous recovery. In contrast, older patients may develop persistent SSS due to pre-existing age-related comorbidities, such as age-related conduction system disorders and degenerative fibrosis, often necessitating PPM implantation.^5^ Case reports support this, with studies documenting younger COVID-19–induced SSS patients recovering without PPM placement. For instance, in a case series by Surabova et al., three out of five SSS patients were <50 years of age and were followed up for 30 ± 7.5 months, during which SSS resolved spontaneously without the need for PPM implantation.^17^ Similarly, Powell et al. described a 47-year-old woman who developed SND during acute COVID-19, but her symptoms fully resolved after recovery.^18^ These findings further support that SSS in younger patients post–COVID-19 may often be transient, leading to a lower PPM implantation rate in this subgroup. The unchanged PPM implantation rates in younger patients, despite a rise in SSS incidence, may be due to a higher implanter threshold in otherwise healthy individuals or suggest a transient dysautonomia-mediated bradyarrhythmia that resolves over time. Further longitudinal studies are required to clarify the clinical course and device utilization in this subgroup.

A key strength of this study is the use of a large and diverse patient cohort, enhancing the generalizability of our findings. The dataset, sourced from multiple health care organizations within the TriNetX Research Network, provides a comprehensive assessment of SSS trends across various demographic and clinical subgroups. Moreover, the use of ITS analysis allowed us to evaluate the impact of the COVID-19 pandemic on SSS incidence, while accounting for pre-existing trends. This method is particularly well suited for assessing the effects of major public health disruptions,^19^ such as the COVID-19 pandemic, on disease outcomes. Additionally, our subgroup analysis of patients <50 years of age offers novel insights into age-related differences in SSS trends and PPM implantation rates, contributing valuable information to the current understanding of COVID-19–induced cardiac arrhythmias.

### Limitations

Despite these strengths, there are several limitations to consider. First, while ITS analysis is robust in detecting temporal changes over time, it cannot fully control for all potential confounders, such as changes in health care utilization, coding practices, or external factors influencing SSS diagnosis. Second, the study relies on electronic health records, which introduces the possibility of heterogeneity in diagnostic coding across institutions. Variations in SSS classification and documentation practices could impact the consistency of case identification. Additionally, the dataset lacked detailed information on pacing dependency after device implantation, timing of SSS diagnosis relative to COVID-19 infection, or symptom profiles such as those associated with long-hauler syndrome or dysautonomia, which limits mechanistic inference. Finally, information on disease severity was not available in our dataset, limiting our ability to determine whether post–COVID-19 PPM placement trends were influenced by changes in physician decision-making or evolving treatment protocols.

## Conclusion

This study demonstrates a significant increase in the incidence of SSS following the onset of the COVID-19 pandemic, with a corresponding rise in PPM implantation in the overall SSS population. However, in patients <50 years of age, while SSS incidence increased significantly post–COVID-19, PPM implantation rates remained unchanged, suggesting a more transient and milder nature of SSS in younger individuals. These findings highlight SARS-CoV-2 as a potential trigger for SSS, reinforcing the need for ongoing cardiovascular monitoring in affected patients. Future research should explore the long-term impact of COVID-19 on cardiac conduction abnormalities and refine age-specific management strategies for SSS in the post–COVID-19 era.
